# FEM-Based Wave Propagation Modelling for SHM: Certain Numerical Issues in 1D Structures

**DOI:** 10.3390/ma13092051

**Published:** 2020-04-28

**Authors:** Magdalena Palacz, Arkadiusz Żak, Marek Krawczuk

**Affiliations:** Department of Mechatronics and High Voltage Engineering, Faculty of Electrical and Control Engineering, Gdańsk University of Technology, Narutowicza 11/12, 80-233 Gdańsk, Poland

**Keywords:** wave propagation, damage detection, numerical modelling

## Abstract

The numerical modelling of structural elements is an important aspect of modern diagnostic systems. However, the process of numerical implementation requires advanced levels of consideration of multiple aspects. Important issues of that process are the positive and negative aspects of the methods applied. Therefore the aim of this article is to familiarise the reader with the most important aspects related to the process of numerical modelling of one-dimensional problems related to the phenomena of the propagation of elastic waves and their application for damage detection purposes.

## 1. Introduction

**Figure 1:**
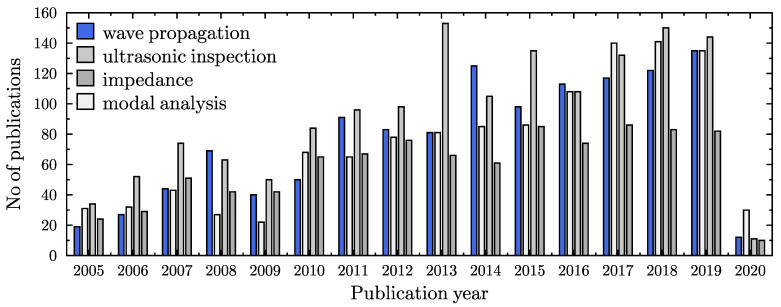
Graphic summary of the number of publications, related with structural parameters used for structural health monitoring (SHM) systems. Web of Science (26 March 2020).

**Figure 2:**
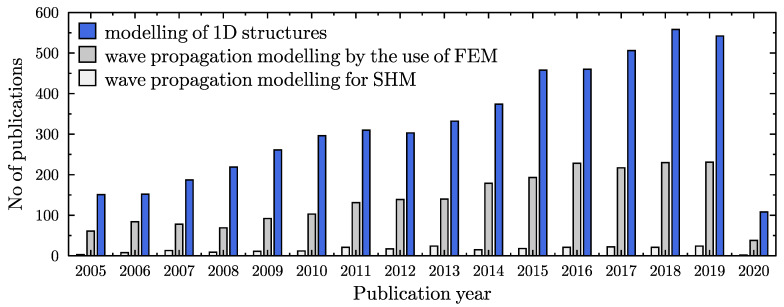
Graphic summary of the number of publications, related with modelling of 1D elements. Web of Science (26 March 2020).

Structural condition monitoring has been a subject of great interest for many leading research groups for last several decades. It has also become more and more popular among engineers, who put a lot of effort into the reduction of maintenance costs [1]. As technical data for further analysis, various structural parameters and their changes have been considered in the case of damage detection algorithms [2,3]. This includes structural parameters obtained from: modal analysis [4,5,6,7,8,9], impedance measurements [10,11], ultrasonic inspection [12,13,14,15], wave propagation [16,17,18,19,20,21,22,23,24,25,26,27,28,29,30], and many others [31,32,33,34,35]. A simple graph is presented in Figure 1 to illustrate in numerals the most popular parameters used in damage detection algorithms. One can see that modal analysis and wave propagation are *the leaders* among many scientific and industrial interests, and the number of publications devoted to these two subjects is relatively large in comparison to ultrasonic inspection and impedance based algorithms. In the authors’ opinion, the simplicity of measurements, equipment availability, and sensitivity to structural changes are the reasons for this *popularity*. Nowadays, numerical calculations based on customised numerical/computational models are ideal alternatives to actual measurements. According to the literature, numerical modelling may be considered as one of the most important aspects of structural condition monitoring [36,37]. In the following figure, Figure 2, a summary of the number of publications devoted to numerical modelling of one-dimensional structural elements, devoted to modelling of wave propagation phenomena, and finally devoted to the use of various numerical models for SHM (structural health monitoring) purposes, has been presented. In every case the number of publications increases, which is definitely a sign that the subject of this paper is of interest to the scientific society.

The purpose of this article is to draw the attention of the research community to some aspects of numerical modelling that may have a significant impact on the correctness of the results of numerical computations; i.e., aspects that may lead to significant errors and misinterpretation of results. The aim of the article is also to draw attention to the problems of numerical nature, which are immanent to modelling. The authors illustrate this in the case of simple one-dimensional structural elements (rods and beams) for various discretisation methods known from the literature.

## 2. Technical Background

As mentioned in Introduction, proper numerical modelling of designed structures has become, nowadays, a key aspect of modern engineering, and it usually covers the total product life cycle starting from its design, current state analysis, and damage prognosis for life-time assessment. Changes in propagating waves have been one of the most popular physical measures used for damage detection purposes [38,39,40,41,42,43,44]. For their proper numerical analysis it is extremely important to employ reliable numerical tools [45,46,47,48,49,50]. For modelling of the wave propagation phenomenon, various commonly known computer methods have been in use. However, the classical finite element method (FEM) may be numerically inefficient, since in the process of wave propagation many higher order vibrational modes participate in the motion. For this reason very dense FE meshes, resulting from very small wavelengths participating in vibrational movements, should be applied because the size of FEs should be comparable to the wavelength of the shortest signal component. This procedure significantly increases the computational size of analysed problems, and directs researchers to other, more efficient numerical methods [51,52,53]. At this point, we would like to strongly emphasise that in the current paper we do not discuss the numerical consequences of taking into account such physical phenomena as wave dispersion and/or attenuation, as we instead concentrate on numerical properties of particular computational approaches.

For several decades, for wave propagation modelling purposes, the time domain spectral finite element method (TDSFEM), proposed by Patera [54], has been successfully applied [36,38,45,55,56,57,58,59,60,61]. It is a computational technique, which combines the properties of approximating polynomials of spectral methods and the same approach as the FEM to divide the analysed area into finite elements. The finite element properties of the method are represented by the fact that for every simple geometrical object, particular points (called nodes) with certain approximating functions (called shape functions or node functions) are defined. These functions describe the distributions of analysed physical quantities within elements and along their boundaries. Non-uniform distribution of nodes within a single element is the result of the distance between zeros of certain polynomials. This allows one to avoid the Runge phenomenon; i.e., large oscillations of approximating polynomials near the structural/element edges [62]. This fact is central for employing high-order polynomials in this method, which is impossible in the case of equally distributed nodes in classical finite element methods [36].

The process of building and solving a numerical model by the use of TDSFEM is similar to FEM and consists of the following steps:

- Division of the analysed structure into a finite number of geometrically simple elements, called spectral finite elements with a certain number of characteristic points called nodes. The spectral finite elements are connected together in a finite number of nodes located at their edges. The number of nodes in the element indicates a selection of the function used for description of the distribution of the physical quantities within the spectral finite elements, depending on their node values. These functions are called node functions or shape functions—Lobatto, Chebyshew, or Laguerre polynomials.
- Transformation of the ordinary or differential equations describing the analysed physical phenomenon to equations of the spectral finite element method. This transformation may be a weak formulation of the method, where there is a weighted residual method applied or a strong formulation, where there the method of minimising the variation functional of the phenomenon is applied. The aforementioned equations, being the problem description, are composed at the level of individual elements and are called local equations, whereas the transformations mentioned correspond to the characteristic matrices of the elements, which are derived. At this step the element matrices are aggregated to form the global characteristic matrices.
- Implementation of boundary conditions.
- Starting the solution process with the appropriate numerical method, leading to obtaining values of sought physical quantities in nodes of individual elements.

For more detailed information about TDSFEM, procedures can be found in [36]. Implementation of TDSFEM with numerous examples of sophisticated models applied to different structural elements have been presented by [42], wherein numerical models for wave propagation with dispersion effects have been described. The models have been experimentally verified and applied for damage detection analysis.

## 3. Numerical Considerations

In order to study the reliability of numerical models applied for wave propagation modelling, various computational investigations have been carried out. Changes in parameters, such as their accuracy or convergence, have been analysed, as they may affect the results of such simulations directly.

### 3.1. Rod Structure

**Figure 3:**
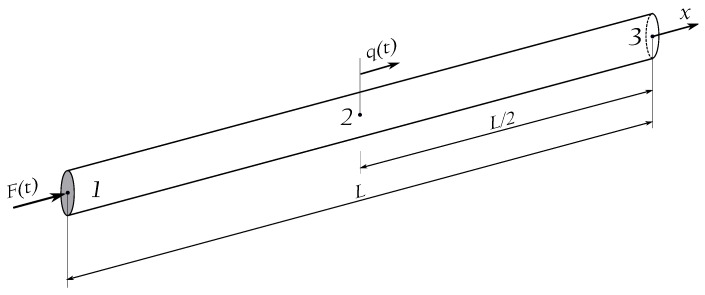
Geometry of a uniform aluminium rod.

As a numerical example, a uniform structural element of a circular cross-section has been selected, as presented in Figure 3. It has been assumed that the length of the element is L=2000 mm, while its diameter is d=50 mm. It has also been assumed that the element is made out of aluminium alloy (elastic modulus E=67.5 GPa, Poisson ratio ν=0.33, material density ρ=2700 kg/m${}^{3}$). It has been assumed that the element is modelled by the use of elementary rod theory. For this type of element, the analytical solutions are commonly known. Therefore, it was easy to validate the correctness of the determined dynamic parameters; i.e., the natural frequencies and modes of natural vibrations. Several cases has been analysed for different approximation functions, in particular:

- Two-node element, Chebyshev polynomial, p=1, 300 finite elements, 301 DOFs;
- Four-node element, Chebyshev polynomial, p=3, 100 finite elements, 301 DOFs;
- Six-node element, Chebyshev polynomial, p=5, 60 finite elements, 301 DOFs;
- Two-node element, Hermite polynomial, p=3, 150 finite elements, 302 DOFs;
- Two-node element, Hermite polynomial, p=5, 100 finite elements, 303 DOFs;
- B-spline polynomial, p=3, 300 nodes, 302 DOFs.

It should be stressed that the application of Hermite and B-spline approximation polynomials enforces the continuity of the strain and stress fields. In the case of Hermite polynomials of the third degree, p=3, this only comprises the first derivatives of the displacement fields (i.e., the strain and stress fields remain continuous but not necessarily smooth). In the case of Hermite polynomials of the fifth degree, p=5, and for B-spline approximation polynomials of the third degree, p=3; the first and second derivatives of the displacement fields are continuous (i.e., strain and stress fields remain continuous and smooth). It should be also added that in the the case of numerical analysis, three different types of boundary conditions have been employed. Firstly, for the analysis of natural frequencies and mode shapes, the clamped-clamped boundary conditions for rods and simply-supported boundary conditions for beams have been used. Secondly, for the analysis of wave propagation, the free–free type of boundary conditions have always been in use, despite the type of structural element.

**Figure 4:**
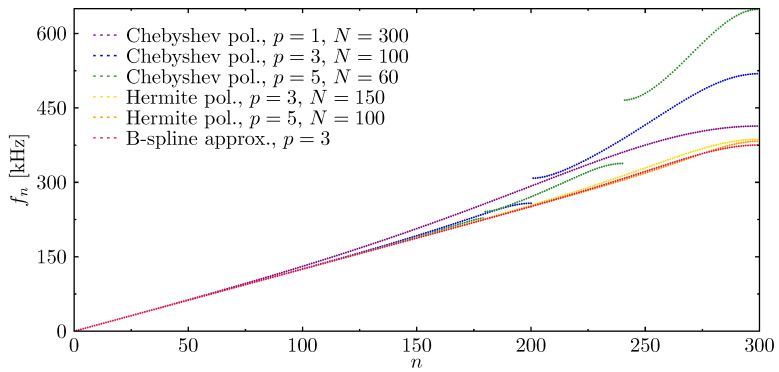
Natural frequencies of the rod calculated based on different approximation polynomials, in the case of the rof with fixed ends.

Computational analysis of structural dynamics requires appropriate modelling of dynamic responses, which are directly linked with accurate modelling of structural natural frequencies and mode shapes. Figure 4 shows the results of numerical calculations of natural frequencies for the same rod (Figure 3) modelled by the listed numerical models. The letter *N* stands for the number of elements used in each analysed case. On the basis of the results presented, it may be concluded that some numerical models show certain discontinuities in their frequency spectra. This phenomenon is caused by the discontinuity of strain and stress fields expressed by spatial derivatives of the approximation functions [63]. That is particularly well visible in the case of Chebyshev approximation polynomials; however, the frequency spectra related to the use of Hermite or B-spline approximation polynomials seem free of such discontinuities.

**Figure 5:**
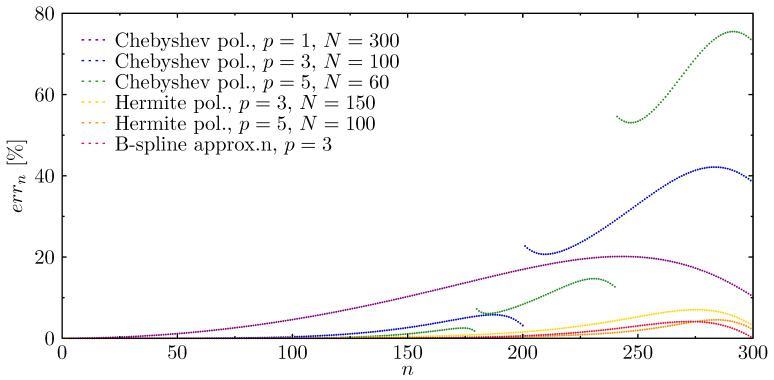
Relative errors of natural frequencies of the rod calculations based on different approximation polynomials, in the case of the rof with fixed ends.

In order to determine the extent to which the presence of such frequency gaps influence the correctness of the results obtained, the following diagrams (Figure 5) have been presented to illustrate the changes in the relative error of the rod natural frequency spectra. It is clearly seen that the use of a higher approximation Chebyshev polynomial results in a significant increase in the relative errors of frequency spectra corresponding to higher frequencies. On the other hand, for the same order of approximation polynomials, but in the case of Hermite polynomials, results in the increase of the relative error are on a level comparable to the error present in calculations obtained by the use of other approximation functions. Moreover, a small change in the relative frequency error may be noticed for all approximation functions analysed. The value of this error seems to be dependent not only on the smoothness of the approximation polynomials used, but the type of node distribution: non-equidistant (Chebyshev p=3 or Chebyshev p=5) or equidistant (Hermite p=3, Hermite p=5, or B-spline p=3).

**Figure 6:**
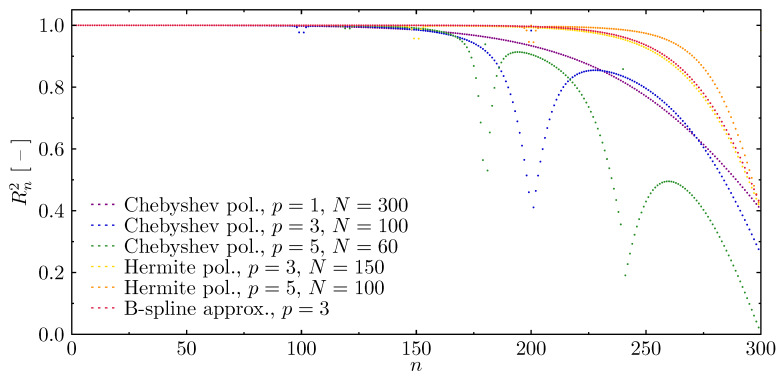
Fitness of natural vibration modes of the rod calculated based on different approximation polynomials, in the case of the rof with fixed ends.

In the analysis of the dynamics of the examined structure, properly represented modes of natural vibrations, obtained numerically, are of great importance. In order to verify the correctness of the results obtained by the proposed numerical models, the errors in the representations of calculated modes of natural vibrations have been determined in comparison to the modes known from analytical solutions for the elementary rod theory, as seen in Figure 6. The fitness value equal to 1 indicates the maximum degree of fitness (i.e., the coefficient of determination) of the determined modes of natural vibrations, while values smaller than 1 indicate inaccurate fitness. It can be seen from the diagrams presented in Figure 6 that in each case considered, the fitness decreases for higher frequencies. Moreover, there are such approximation polynomials (i.e., Chebyshev and Hermite), for which there are strict boundaries of total incompatibility of the determined modes. These discontinuities appear at natural frequency numbers that are multiples of the numbers of finite elements of numerical models [63]. Based on the results obtained, it can be concluded that the correctness of numerical calculations in the dynamics of the rod under investigation is significantly determined by the type of approximation polynomials. The appearance of the first frequency gap limits the usable part of the frequency spectrum in a much more significant manner than the observable decrease of the fitness for higher frequencies. Only in the cases of Chebyshev polynomials of the first degree (linear shape functions) p=1, and B-spline approximation polynomials of the third degree p=3, do the fitness lines remain smooth. In the first case out of these two, however, the values of fitness decrease for higher frequencies much faster; thus, the usable part of the frequency spectrum is smaller.

#### Wave Propagation Analysis

Changes in the propagation of elastic waves have been successfully used in various systems for assessing the technical conditions of mechanical structures. For that purpose, numerical simulations may be an alternative method with which to avoid unexpected costs of maintenance. Modelling of elastic wave propagation requires the use of numerical models, which ensure the correct representation of the structure dynamics, especially for higher frequencies, in order to avoid any signal losses or distortions. This is why scientific research on the development of numerical methods that enable the analysis of this phenomenon has been very popular among various groups in the world.

**Figure 7:**
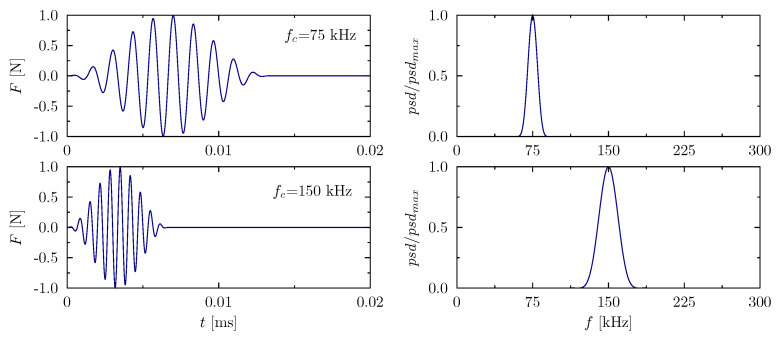
Excitation signals for two different carrier frequencies $f_{c}$ in the time domain (**left**) and their power density spectra in the frequency domain (**right**).

For this purpose, simulations have been carried out to verify the developed numerical models in regard to their application to the analysis of the elastic wave propagation phenomenon. Similar to the described analysis of dynamic parameters, rod models have been developed by the use of elementary theory and the same approximation polynomials have been studied. An example of analysed excitation signals is shown in Figure 7, which illustrates the time signals and their normalised power spectral densities (psd). In both cases (the carrier frequency $f_{c}=75$ kHz and $f_{c}=150$ kHz) it was a sinusoidal signal modulated by a Hanning window. The signal carrier frequencies $f_{c}$ have been selected to visualise the effect of the periodicity of various numerical models on the correctness of the results obtained. The value of the carrier frequency $f_{c}=75$ kHz is outside any spectrum discontinuities, whereas the carrier frequency $f_{c}=150$ kHz is located near such a discontinuity. It should be also noticed that in each case of the excitation signals, a certain range of frequencies $<f_{c}-2f_{m},f_{c}+2f_{m}>$ ($f_{m}$—modulation frequency equal to $f_{c}/m$, *m*—modulation equal to 10) has been simulated. For the carrier frequency of 75 kHz signal excited frequencies fall into the frequency range starting from 60 kHz and ending at 90 kHz, whereas in the case of the carrier frequency of 150 kHz, signal excited frequencies fall into the frequency range starting from 120 kHz and ending at 180 kHz. In both cases, the excitation amplitude was 1 N. Figure 7 shows the time and power spectra of the excitation signals.

**Figure 8:**
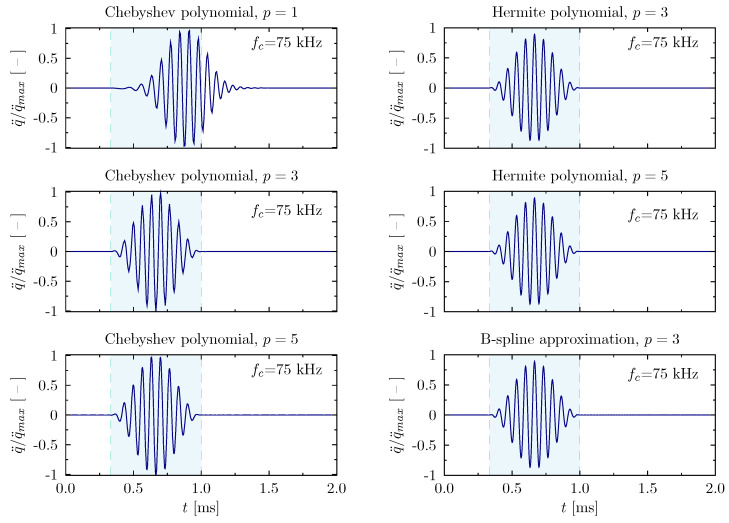
Wave propagation patterns of the rod calculated based on different approximation polynomials for the carrier frequency $f_{c}=75$ kHz, in the case of the rod of free ends.

In the following figures the examples of patters of propagating elastic waves have been shown. Thus, Figure 8 illustrates the changes registered for the rod under investigation in the case in which the rod is excited by a signal of the carrier frequency of 75 kHz. Illustrated signals have been registered at the excited end of the rod. The time of analysis has been set to 0.8 $ms+2/f_{m}$ and it was divided into $2^{13}$ equal time steps. For each signal, an appropriate time window is marked, within which the propagating wave packet should be located at the end of the analysis. As a solution method for the equations of motion, the Newmark method has been chosen (α=0.5, β=0.25). Material damping has been neglected. It is worth mentioning that the selected carrier frequency of 75 kHz is beyond the boundaries of frequency spectrum discontinuity areas. As it can be noticed in the presented figure, the obtained patterns of propagating elastic waves, except the one obtained for Chebyshev polynomials of the first order p=1, seem to be correct; i.e., there is no visible influence of the periodicity of numerical models used on the representation of the calculated patterns of elastic waves.

**Figure 9:**
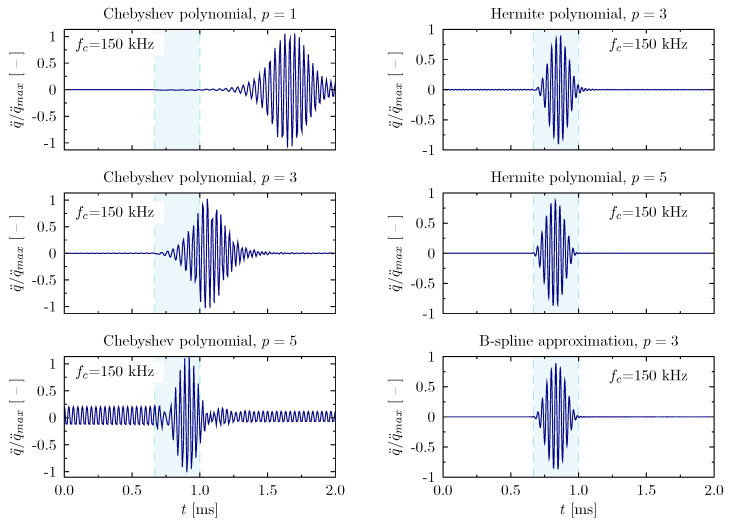
Wave propagation patterns of the rod calculated based on different approximation polynomials for the carrier frequency $f_{c}=150$ kHz, in the case of the rod of free ends.

The next example (Figure 9) demonstrates similar changes, but registered for the excitation signal of the carrier frequency equal to 150 kHz. This carrier frequency is located close to the frequency band only slightly visible in the diagrams in Figure 5 and Figure 6. A narrower time window is directly related to the shorter excitation time. In the discussed case it can be clearly noticed that a higher frequency of the excitation signal definitely requires reliable numerical models. In all of the numerical models based on the use of Chebyshev approximation polynomials, the registered signals have fallen outside the time window. One can see at least two examples of the registered signals, which may indicate the discursive nature of the wave propagation phenomena. However, this is very much misleading because the applied rod theory is non-dispersive [63]. The signals obtained by the use of numerical models based on the other approximation polynomials are represented in a correct manner.

### 3.2. Beam Structure

It should be emphasised that the results of numerical investigations presented so far have been directly related to the propagation of longitudinal elastic waves in rod structural elements. However, the conclusions drawn can be easily generalised and extended onto other types of structural elements, such as beams, plates, shells, or solid elements. As the next numerical example, a beam of a uniform cross-section has been selected, as presented in Figure 3. All geometrical and material properties, as well as the form and type of boundary conditions and excitation signals were the same as in the case of the analysed rod element. The modelled beam has been divided into the same number of spectral finite elements. The elementary theory for beams has been employed in this analysis.

**Figure 10:**
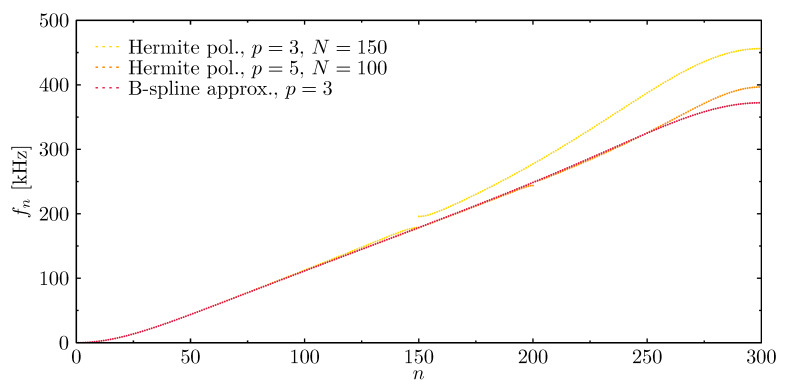
Natural frequencies of the beam calculated based on different approximation polynomials, in the case of a beam with simply-supported ends.

Figure 10 presents the results of natural frequency calculations of the beam modelled by means of several numerical models using different approximation polynomials: Hermite polynomials of the third order p=3, Hermite polynomials of the fifth order p=5, and B-spline approximation polynomials of the third order p=3. Again, the letter *N* stands for the number of finite elements used in each analysed case. On the basis of the results presented, it may be concluded that numerical models based on Hermite approximation polynomials show certain discontinuities in their frequency spectra. This phenomenon is particularly visible near the boundaries of frequency bands related to the total number of finite elements used in the analysis. As before, these are the so-called frequency band gaps and their appearance is a direct proof of the periodical nature of the numerical models under investigation [63].

**Figure 11:**
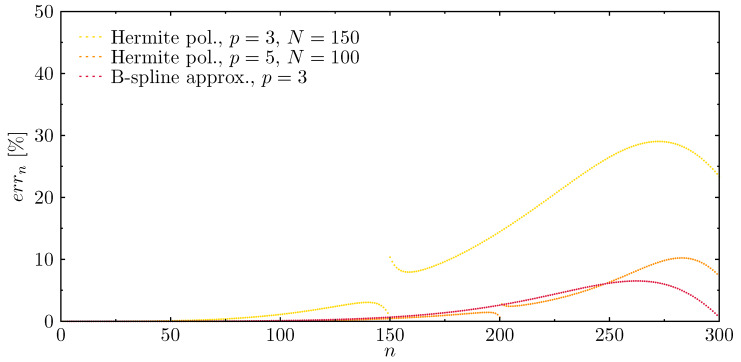
Relative errors of natural frequencies of the beam calculated based on different approximation polynomials, in the case of a beam with simply-supported ends.

In order to determine the extent to which such frequency gaps influence the correctness of the results obtained, the following diagrams (Figure 11) have been presented to illustrate changes in the relative error of the beam’s natural frequency spectrum. It is clearly seen that Hermite polynomials of the third order p=3 result in significant increases in the relative errors in the parts of the frequency spectra corresponding to higher natural frequencies. On the other hand, Hermite polynomials of the fifth degree p=5 result in an increase in the relative error that reaches only 10%. Moreover, the minimal changes in the relative frequency error are noticed for B-spline approximation, for the third degree of approximation polynomials p=3. It can be summarised that the nature of the chances in the relative frequency error appears as independent of the order of approximation polynomials or node distribution—non-equidistant or equidistant—but it is dependent on the order of the smoothness of the displacement field in relation to the order of approximation polynomials.

**Figure 12:**
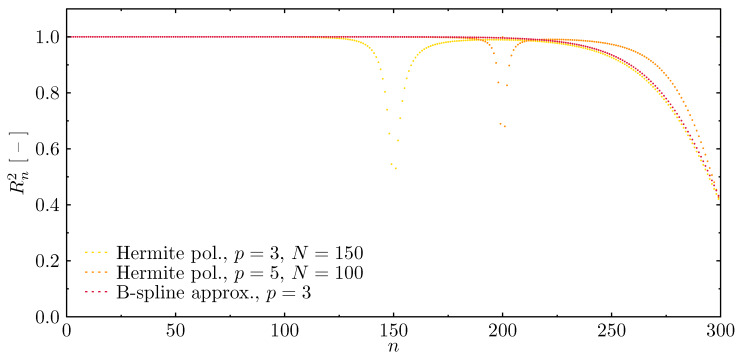
Fitness of natural vibration modes of the beam calculated based on different approximation polynomials, in the case of a beam with simply-supported ends.

Additionally, in the case of beam elements, in order to verify the correctness of the results achieved by the proposed numerical models, errors in the representation of the mode shapes have been determined (Figure 12). Again, the fitness value equal to 1 indicates the maximum degree of fitness (i.e., the coefficient of determination) of the determined modes of natural vibrations, while values smaller than 1 indicate inaccurate fitness.

It can be seen from the diagrams presented in Figure 12 that in each case considered the fitness decreases for higher frequencies. Moreover, in both Hermite approximation polynomials there are are strict boundaries of total incompatibility of the determined modes. These discontinuities appear at natural frequency numbers that are multiples of the number of finite elements of numerical models. As before, in the case of the beam under investigation it should be emphasised that the correctness of numerical calculations is significantly determined by both the number of finite elements and the degree of the polynomial approximation function, as these parameters determine the extent of the usable part of the frequency spectrum that is up as the first frequency band gap. Only in the case of B-spline approximation polynomials of the third degree p=3 are there no visible frequency band gaps in the frequency spectra, and the line representing fitness of the calculated modes remains smooth. Additionally, in this case the usable part of the frequency spectrum is the greatest.

#### Wave Propagation Analysis

**Figure 13:**
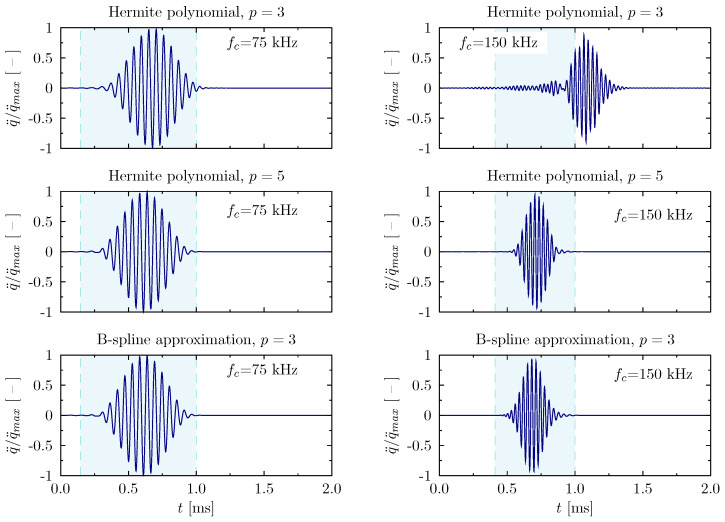
Wave propagation patterns of the beam calculated based on different approximation polynomials for the carrier frequencies $f_{c}=75$ kHz and $f_{c}=150$ kHz, in the case of a beam with free ends.

In order to illustrate the changes in the patterns of propagating flexural waves in the aluminium beam element, certain results have already been presented in Figure 13. The excitation signals used have been presented in Figure 7. Similarly to the examples discussed above related to the wave propagation analysis in the rod element, the signal carrier frequencies $f_{c}$ have been selected to take into account the effect of the periodicity of the numerical model on the correctness of the results obtained. Since the group and the phase velocities are different in the case of the elementary theory of the beam employed by the authors, some signal dispersion should be observed. As a consequence, the group speed of propagating waves also depends on the frequency, as it is equal to $c_{g}=4854.1$ m/s for $f_{c}$ = 150 kHz and $c_{g}=3432.3$ m/s for $f_{c}$ = 75 kHz. Therefore the time of the analysis have been adjusted accordingly in each particular case.

The last of the presented diagrams illustrates the changes in the patters of propagating waves in relation to the type of assumed approximation polynomials employed by numerical modes of the beam. The results presented in Figure 13 concern both excitation frequencies $f_{c}=75$ and $f_{c}=150$ kHz. Based on the results obtained, it can be concluded that in the case of the carrier frequency of 75 kHz the result fits quite well to the selected time window. On the other hand, in the case of the analysis carried out for the signal of the carrier frequency equal to 150 kHz, the influence of the periodic nature of the numerical model on the correctness of the obtained results is clearly visible. Signal frequency components near the frequency band gap are proportionally misrepresented in both aspects: their propagation speed related to increased values of natural frequencies and distorted shapes of modes of natural vibrations.

## 4. Discussion and Conclusions

The numerical studies carried out by the authors concerned dynamic responses of simple one-dimensional structures. The analysed responses covered natural frequencies and modes of natural vibrations, and the accuracy of their representations. The study aimed at the investigation of the influence of this accuracy on wave propagation responses in two one-dimensional structures, those being: a rod modelled according to the elementary theory of rods and a beam modelled according to the elementary theory of beams. Despite the fact that those structural elements are one-dimensional and the employed theories of their dynamic behaviour are the simplest available, the conclusions presented below remain valid for other structural elements. They also apply to two and three-dimensional structural elements of complex geometries and material properties as long as their numerical representations have the features typical of periodic structures, i.e., characterised by a large number of finite elements of the same or similar size, which is a typical for wave propagation analysis.

The conclusions based on the results obtained by the authors and presented in the current work can be summarised in the following way:

- Numerical models based on the use of finite elements may be thought of as representing periodic structures of certain properties as long as the they include a large number of finite elements of the same or similar size.
- The periodic nature of these models results from the level of the discontinuity of the displacement fields and the order of approximation polynomials employed to build appropriate finite elements, and manifests by the presence of so-called frequency band gaps in calculated frequency spectra.
- As a result of that, the frequency spectra are divided into a number of regions separated by these frequency band gaps, which effectively limits the usable parts of available frequency spectra, based on which the calculated dynamics responses remain unaffected by the periodic nature of discrete numerical models used.
- The number of regions is correlated with the order of approximation polynomials and the level of continuity of the displacement field (1 corresponds to the continuity of the displacement field, 2 to the continuity of the strain and stress fields, and 3 to the continuity of their derivatives).
- The biggest number of such regions was observed in the case of Chebyshev approximation polynomials and the continuity of the displacement field only, which is typical for classical FEM and TDSFEM. The only exceptions are the approximation polynomials of the first degree p=1, where there are no visible frequency band gaps in the calculated spectrum and where the order of approximation polynomials is the same as the level of the continuity of the displacement field. However, the application of such approximation polynomials is characterised by the greatest average errors.
- Application of other types of approximation polynomials, such as Hermite polynomials, leads to smooth frequency spectra only in the case of rods. For calculated beam elements, the use of Hermite approximation polynomials does not improve the situation. In their cases, the orders of approximation polynomials p=3 and p=5 are greater than the level of the continuity of the displacement fields, which are equal to 2 and 3, respectively.
- Only in the case of B-spline approximation polynomials of the third degree p=3 is the observed behaviour different, and the calculated characteristics remain smooth. In this case the order of approximation polynomials is equal to the level of the continuity of the displacement field. As a result the entire spectrum is free of frequency band gaps.
- The influence of frequency band gaps is typically associated to the upper part of the calculated frequency spectra; however, it may significantly influence the representation of modal responses in much lower parts of these spectra.
- This influence may lead to significant numerical errors, as a result of which calculated wave propagation responses may be misrepresented and possess artificial features; for example, they may suggest the presence of strong damping or dispersion.
- As a consequence it is strongly recommended by the authors, prior any wave propagation analysis, that one performs a thorough analysis of natural frequencies and modes of natural vibrations in order to recognise the regions affected by the periodicity of numerical models employed.
- The features discussed in this paper may even more profoundly influence dynamic responses of two-dimensional and three-dimensional structures. This is the subject of the authors’ future research.

## Abbreviations

The following abbreviations are used in this manuscript:

SHM	Structural health monitoring
FEM	Finite element method
TDSFEM	Time domain spectral finite element method
